# Der DMKG-Kopfschmerzfragebogen: Ein neues Tool der Deutschen Migräne- und Kopfschmerzgesellschaft zur Verbesserung der Kopfschmerzversorgung

**DOI:** 10.1007/s00482-025-00870-0

**Published:** 2025-02-13

**Authors:** Ruth Ruscheweyh, Charly Gaul, Thomas Dresler, Tim Patrick Jürgens, David Lewis, Torsten Kraya, Lars Neeb, Victoria Ruschil, Gudrun Goßrau

**Affiliations:** 1 Kopfschmerzambulanz, Neurologische Klinik und Poliklinik, Klinikum der LMU München, Marchioninistr. 15, 81377 München, Deutschland; 2 Kopfschmerzzentrum Frankfurt, Frankfurt, Deutschland; 3 Klinik für Psychiatrie und Psychotherapie, Universitätsklinikum Tübingen, Tübingen, Deutschland; 4 LEAD Graduiertenschule & Forschungsnetzwerk, Universität Tübingen, Tübingen, Deutschland; 5 Deutsches Zentrum für Psychische Gesundheit (DZPG), Standort Tübingen, Tübingen, Deutschland; 6 Kopfschmerzzentrum Nordost, Neurologische Klinik und Poliklinik, Universitätsklinik Rostock, Rostock, Deutschland; 7 Neurologische Klinik, KMG Krankenhaus Güstrow, Güstrow, Deutschland; 8 LEWIS Neurologie, Stuttgart, Deutschland; 9 Neurologische Klinik, Krankenhaus Sankt Georg Leipzig, Leipzig, Deutschland; 10 Klinik für Neurologie, Universitätsklinikum Brandenburg an der Havel GmbH, Brandenburg an der Havel, Deutschland; 11 Abteilung Neurologie mit Schwerpunkt Epileptologie, Universitätsklinikum Tübingen, Tübingen, Deutschland; 12 Zentrum für seltene Erkrankungen, Universitätsklinikum Tübingen, Tübingen, Deutschland; 13 Kopfschmerzambulanz, Universitätsschmerzcentrum, Medizinische Fakultät der TU Dresden, Universitätsklinikum Dresden, Dresden, Deutschland

**Keywords:** Kopfschmerzversorgung, Migräne, Kopfschmerz vom Spannungstyp, Clusterkopfschmerz, Anamnese, Headache care, Migraine, Tension type headache, Cluster headache, Medical history taking

## Abstract

Für die Diagnosestellung, Therapie und Verlaufsbeurteilung von Kopfschmerzerkrankungen müssen in der Anamnese zahlreiche Informationen erfragt werden. Dies wird wesentlich erleichtert, wenn die Patient:innen diese Informationen bereits vorab anhand eines Fragebogens zusammenstellen. Expert:innen der Deutschen Migräne- und Kopfschmerzgesellschaft (DMKG) haben dafür den DMKG-Kopfschmerzfragebogen entwickelt, der sowohl kopfschmerzspezifische Informationen (Häufigkeit, Charakteristika, Begleitsymptome, Medikation) als auch Begleiterkrankungen erfasst und darüber hinaus validierte Skalen zu psychischen Begleitfaktoren, Beeinträchtigung und Lebensqualität enthält. Es gibt separate Versionen für Erst- und Wiedervorstellung sowie ein Clusterkopfschmerzmodul. Der DMKG-Kopfschmerzfragebogen ist für Fachkreise nach Registrierung online frei erhältlich (https://www.dmkg.de/dmkg-kopfschmerzfragebogen) und steht zum Ausdrucken oder als ausfüllbares PDF zur Verfügung. Er kann mit dem enthaltenen PDF-Kopfschmerzkalender oder mit der DMKG-App als elektronischer Kopfschmerzkalender kombiniert werden. Eine digitale Version ist geplant. Der DMKG-Kopfschmerzfragebogen ist ein wichtiger Baustein zur Qualitätssicherung der Kopfschmerzversorgung in Deutschland.

## Abstract (en)

To enable state-of-the-art diagnosis, therapy and monitoring of headache disorders, comprehensive history taking is necessary and can be time-consuming. This is facilitated substantially if the patient gathers the needed information beforehand, using a specialized questionnaire. To this end, experts of the German Migraine and Headache Society (DMKG) have developed the DMKG headache questionnaire. It assesses central information such as headache frequency, characteristics, accompanying symptoms and medication, as well as comorbidities and validated scales for psychological cofactors, disability and quality of life. The complete version for a first visit is complemented by a shorter version for follow-up visits and a specialized cluster headache module. For headache care professionals, the DMKG headache questionnaire is free after registration (https://www.dmkg.de/dmkg-kopfschmerzfragebogen). It is available for a printout or as fillable PDF and can be combined with the included PDF headache diary or with the DMKG-App (smartphone headache diary). A digital version is in the planning. The DMKG headache questionnaire is an important step towards quality assurance of headache care in Germany.

## Einleitung

Kopfschmerzen gehören zu den häufigsten Gründen für hausärztliche, neurologische oder schmerztherapeutische Konsultationen. In der Akutsituation liegt der Fokus auf dem Ausschluss sekundärer Ursachen. In den meisten Fällen handelt es sich aber um bereits länger bestehende, primäre Kopfschmerzerkrankungen, wie z. B. Migräne. In diesen Fällen ist eine gute Kopfschmerzanamnese aufwendig und muss biopsychosoziale Faktoren mitberücksichtigen [1–3]. Neben den Kopfschmerzcharakteristika sind die Kopfschmerztage und Akutmedikationstage pro Monat, bisherige Therapieversuche, aktuelle und frühere Medikamente zur Akuttherapie und Kopfschmerzprophylaxe sowie Lebensumstände und Begleiterkrankungen zu erfragen. Zusätzlich sollten die kopfschmerzbezogene Beeinträchtigung, Einschränkung der Lebensqualität und psychische Begleitfaktoren mittels validierter Skalen erfasst werden. Viele Praxen und Zentren mit speziellem Interesse an Kopfschmerzen haben daher eigene Anamnesebögen, die von Patient:innen vor dem Sprechstundentermin ausgefüllt werden und als Grundlage für das Anamnesegespräch dienen. Ein wesentlicher Vorteil des Ausfüllens eines solchen Fragebogens zu Hause ist, dass die Patient:innen vorbereitet in die Sprechstunde kommen.

## Der DMKG-Kopfschmerzfragebogen

Die Erhebung eines gemeinsamen, standardisierten Basisdatensatzes hätte den Vorteil, die Dokumentation zu vereinheitlichen. Dies würde sowohl eine zentrumsübergreifende Qualitätskontrolle ermöglichen als auch eine Vereinfachung der Versorgungsforschung. Daher hat die Deutsche Migräne- und Kopfschmerzgesellschaft (DMKG) den *DMKG-Kopfschmerzfragebogen* entwickelt, der die wichtigsten Punkte der Kopfschmerzanamnese sowie validierte Skalen zur Erfassung von kopfschmerzbezogener Beeinträchtigung (Migraine Disability Assessment [MIDAS]; [4]), die Angst‑, Depressions- und Stressskala (DASS; [5]), die Bewertung des aktuellen Gesundheitszustands und der gesundheitsbezogenen Lebensqualität und den DMKG-Kopfschmerzkalender enthält. Darüber hinaus unterstützt der Fragebogen die diagnostische Zuordnung primärer Kopfschmerzen nach den Kriterien der Internationalen Kopfschmerzklassifikation (International Classification of Headache Disorders [ICHD-3]; [6]). Es gibt ein *Clusterkopfschmerzzusatzmodul* mit angepassten Fragen, Clusterkopfschmerzkalender und einer validierten Beeinträchtigungsskala (Cluster Headache Impact Questionnaire [CHIQ]; [7]); ein Zusatzmodul für Gesichtsschmerzen ist geplant. Der *DMKG-Verlaufsbogen* dient der Verlaufsanamnese und -beurteilung. Statt mit einem Papierkopfschmerzkalender kann der DMKG-Kopfschmerzfragebogen auch mit der DMKG-App (elektronischer Kopfschmerzkalender, frei verfügbar in den App-Stores) kombiniert werden. Für Patient:innen mit Clusterkopfschmerz gibt es die DMKG Cluster-App. Ein Überblick über den DMKG-Kopfschmerzfragebogen findet sich in Tab. 1, die komplette Version ist für Fachkreise nach Registrierung frei verfügbar unter https://www.dmkg.de/dmkg-kopfschmerzfragebogen. Der DMKG-Kopfschmerzfragebogen wurde erstmals beim Deutschen Schmerzkongress 2024 (16.–19.11. in Mannheim) vorgestellt.

**Tab. 1:** Inhalte des DMKG-Kopfschmerzfragebogens (Überblick)

Überbegriff	Details
*DMKG-Kopfschmerzfragebogen (bei Erstvorstellung) (9 Seiten* *+* *Kopfschmerzkalender)*	
Demografische Daten	Inkl. Gewicht und Größe
Kopfschmerzdaten	Inkl.:
	Beginn der Kopfschmerzen
	Kopfschmerztage/Monat, starke Kopfschmerztage/Monat
	Schmerzmitteltage/Monat
	Kopfschmerzcharakteristika (Lokalisation, Charakter, Begleitsymptome) mit Zuordnung zu möglichen Kopfschmerzdiagnosen nach ICHD-3-Kriterien [6]
	Bisherige Bildgebung des Kopfs
	Familienanamnese
Kopfschmerzbehandlung	Aktuelle und frühere Medikamente mit Gründen für das Absetzen
	Nichtmedikamentöse Verfahren, Neurostimulation
	Stationäre, ambulante oder tagesklinische Schmerztherapien
Validierte Skalen	Depressions‑, Angst- und Stressskala (DASS)
	Lebensqualität: Veterans Rand-12 (VR-12)^1^ oder Bewertung durch Patient:in (auf der NRS)
	Kopfschmerzspezifische Beeinträchtigung: Migraine Disability Assessment (MIDAS)
	Aktueller Gesundheitszustand (NRS)
Komorbiditäten	Begleiterkrankungen
	Begleitmedikation
Berufstätigkeit und sozialmedizinische Daten	Inkl. Arbeitsunfähigkeit, Arbeitslosigkeit, Erwerbsunfähigkeitsrente, Grad der Behinderung
Kopfschmerzkalender	Kopfschmerzstärke, -dauer, -charakteristika, Einnahme der Akutmedikation
	Alternativ zum Papierkalender: Nutzung der DMKG-App und Mitbringen des Ausdrucks
*Zusatzmodul Clusterkopfschmerz (2 Seiten* *+* *Clusterkopfschmerzkalender)*	Attacken/Woche
	Anwendungen Akutmedikation/Woche
	Clusterkopfschmerzcharakteristika
	Beginn und Ende der letzten Episode
	Cluster Headache Impact Questionnaire (CHIQ)
	Clusterkopfschmerzkalender (oder DMKG Cluster-App)
*Verlaufsbogen (6 Seiten* *+* *Kopfschmerzkalender)*	Aktualisierung der oben genannten Daten (soweit veränderlich)
	Zusätzlich: Patient Global Impression of Change (PGIC)

^1^ Nur in der digitalen Version, da händische Auswertung nicht möglich. *NRS* numerische Rating-Skala

## Vorarbeiten, Validierung und Qualitätskontrolle

Die DMKG verwendet seit 2020 im Projekt *DMKG-Kopfschmerzregister* bereits einen (digitalen) Kopfschmerzfragebogen sowie die DMKG-App als elektronischen Kopfschmerzkalender. Der hier vorgestellte DMKG-Kopfschmerzfragebogen ist ein vereinfachter PDF-Auszug des Fragebogens im Kopfschmerzregister, und der im DMKG-Kopfschmerzfragebogen enthaltene Kopfschmerzkalender ist ein Auszug der DMKG-App.

Das Projekt DMKG-Kopfschmerzregister ist im Juni 2020 gestartet (www.kopfschmerzregister.de, [8]). Dort können Patient:innen ihre digital eingegebenen Daten (Kopfschmerzfragebogen, DMKG-App) für Ärzt:innen an teilnehmenden Praxen/Zentren zur Nutzung während des Anamnesegesprächs freigeben. Nach ärztlicher Validierung und Ergänzung erfolgt die Übernahme in eine pseudonymisierte Auswertungsdatenbank. Diese wird für die Versorgungsforschung der DMKG verwendet, woraus bereits mehrere internationale Publikationen entstanden sind [9–11]. Zum Zeitpunkt September 2024 nehmen 35 Zentren am Kopfschmerzregister teil, über 3900 Patient:innen haben mindestens eine abgeschlossene ärztliche Visite, insgesamt liegen Daten von > 11.000 Visiten vor. Die auch separat nutzbare DMKG-App wird deutschlandweit von > 30.000 Patient:innen verwendet. Zur Qualitätskontrolle und Validierung werden regelmäßig Umfragen unter teilnehmenden Patient:innen und Ärzten durchgeführt, deren Ergebnisse somit zumindest teilweise auf den hier vorgestellten DMKG-Kopfschmerzfragebogen mit Kopfschmerzkalender übertragbar sind.

**Abb. 1:**
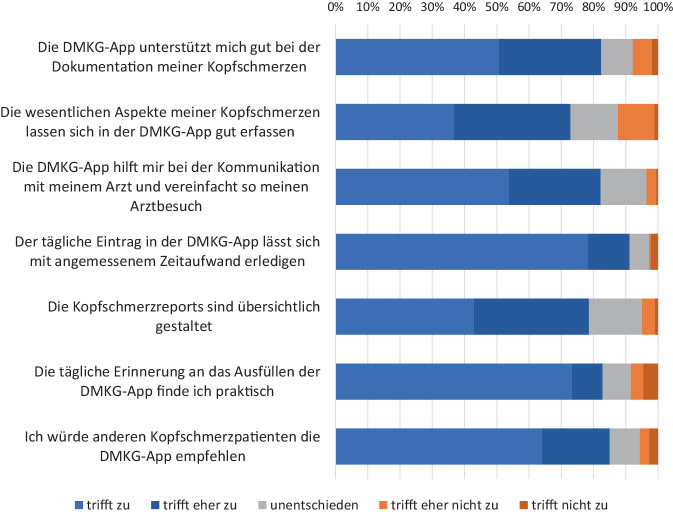
Ergebnisse einer Umfrage unter Nutzern der DMKG-App 2022 (*n* = 602, Auszug). Gezeigt ist für jede Aussage die prozentuale Anzahl der Nutzer, die die jeweilige Bewertung abgegeben haben

An einer anonymen Umfrage unter Nutzern des elektronischen Kopfschmerzkalenders (DMKG-App) im Herbst 2022 nahmen 634 Nutzer teil, 602 beantworteten alle 11 Fragen, und 287 machten Freitextkommentare (https://www.kopfschmerzregister.de/umfragen/). Die Bewertungen waren ganz überwiegend positiv, sowohl bezüglich der Unterstützung bei der Kopfschmerzdokumentation als auch für die Kommunikation mit dem Arzt/der Ärztin und den täglichen Zeitaufwand (siehe Abb. 1). 85 % der Nutzer gaben an, dass sie die DMKG-App weiterempfehlen würden. Die Freitextkommentare enthielten zahlreiche konstruktive Verbesserungsvorschläge, von denen mehrere in der Zwischenzeit bereits umgesetzt wurden (z. B. Ergänzung der Begleitsymptome um die Punkte Schwindel, Geruchsempfindlichkeit, Konzentrationsstörungen und Müdigkeit/Erschöpfung; Integration der Anzahl der Tage mit Akutmedikation direkt in die Kalenderansicht). Im Sommer 2023 wurde eine Umfrage unter teilnehmenden Ärzt:innen durchgeführt, mit 11 Teilnehmer:innen. Auch hier waren die Bewertungen überwiegend positiv, insbesondere stimmten 91 % der Aussage „Das Kopfschmerzregister beinhaltet die Informationen, die ich für meine Kopfschmerzsprechstunde brauche“ zu und 73 % stimmten „Das Kopfschmerzregister vereinfacht meine Arbeit“ zu. Als besonders hilfreich wurde die Anzeige der Kopfschmerzdaten (z. B. Kopfschmerztage im Monat) sowie des Kopfschmerzkalenders angesehen (100 % der Teilnehmer nutzen diese immer oder oft) sowie die Anzeige der Ergebnisse der validierten Skalen, die Dokumentation früherer Kopfschmerzprophylaktika und die Anzeige der Begleiterkrankungen und Begleitmedikation (72 %, 82 %, und 82 % nutzen diese immer oder oft). Alle Teilnehmer gaben an, dass sie Kollegen die Nutzung des Kopfschmerzregisters empfehlen würden. Auch wenn die Anzahl der Teilnehmer an der Umfrage begrenzt war, zeigt dies doch, zusammen mit den bereits > 3900 eingeschlossenen Patienten, die sehr gute Akzeptanz unter den Ärzt:innen.

Die Qualitätskontrolle des Kopfschmerzregisters wird durch regelmäßige Umfragen unter Ärzt:innen und Patient:innen und entsprechende Anpassungen fortgeführt. Geplant ist außerdem eine direkte Validierung des DMKG-Kopfschmerzfragebogens selbst durch Befragung der daran teilnehmenden Ärzt:innen und Patient:innen. Zur Befragung der Patient:innen ist ein Link (inkl. QR-Code) zur Befragung auf dem DMKG-Kopfschmerzfragebogen angebracht, über den laufend Rückmeldungen erhoben werden. Die Befragung teilnehmender Ärzt:innen erfolgt per E‑Mail. Hier soll neben Zufriedenheit, Praktikabilität und Bewertung der einzelnen Komponenten auch die Übereinstimmung zwischen DMKG-Kopfschmerzfragebogen und persönlicher Anamnese bewertet werden.

## Gemeinsamkeiten und Unterschiede zum Deutschen Schmerzfragebogen

Der von der Deutschen Schmerzgesellschaft herausgegebene Deutsche Schmerzfragebogen [12] hat sich als generischer Schmerzfragebogen seit vielen Jahren bewährt und wurde bereits mehrfach überarbeitet. Er kann auch bei Kopfschmerzpatient:innen eingesetzt werden. Allerdings sind bei Kopfschmerzen im Vergleich zu anderen Schmerzerkrankungen einige Besonderheiten zu berücksichtigen:

- Meist attackenartige Manifestation, dazwischen weitgehende Beschwerdefreiheit
- Diagnostisch und therapeutisch bedeutsame Begleitsymptome (z. B. Übelkeit, Erbrechen, Lärm- und Lichtempfindlichkeit, Migräneaura, trigeminoautonome Symptome)
- Die medikamentöse Behandlung unterscheidet zwischen Akuttherapie und Prophylaxe.
- Der Übergebrauch von Akutmedikation kann zur Chronifizierung (Kopfschmerz bei Medikamentenübergebrauch) führen.
- Die Erstattungsfähigkeit moderner Therapien erfordert die detaillierte Dokumentation unwirksamer, unverträglicher oder ungeeigneter Vortherapien.
- Wichtigste Verlaufsparameter sind Kopfschmerztage und Schmerzmitteltage im Monat (Erfassung über Kopfschmerzkalender empfohlen).
- Feste Einbeziehung bestimmter nichtmedikamentöser kopfschmerzprophylaktischer Maßnahmen (Ausdauersport, Entspannungsverfahren, ggf. verhaltenstherapeutische Verfahren [13–15]) in den Therapiealgorithmus
- Im Vergleich zu anderen Schmerzpatient:innen sind Kopfschmerzpatient:innen meist jünger und weniger häufig anhaltend arbeitsunfähig oder berentet.

Die Fokussierung auf Kopfschmerzen erlaubt dem DMKG-Kopfschmerzfragebogen eine genauere Erfassung kopfschmerzspezifischer Punkte bei gleichzeitig überschaubarem Umfang. Wichtige Stärken des DMKG-Kopfschmerzfragebogens sind:

- Erfassung der zentralen Parameter Kopfschmerztage und Tage mit Akutmedikation pro Monat
- Erfassung von Kopfschmerzcharakteristika zur Unterstützung der diagnostischen Zuordnung primärer Kopfschmerzen nach der ICHD‑3 [6]
- Verwendung von kopfschmerzspezifischen Fragebögen (MIDAS [4] und CHIQ [7]), die auch für die Verlaufsbeurteilung relevant sind
- Erfassung der aktuellen und früheren Akutmedikamente und Prophylaxen, inklusive Grund für das Absetzen
- Erfassung der nichtmedikamentösen Prophylaxe
- Integration des DMKG-Kopfschmerzkalenders/Kombinierbarkeit mit der DMKG-App

Auf der anderen Seite gibt es aber auch zahlreiche Gemeinsamkeiten zwischen dem Deutschen Schmerzfragebogen und dem DMKG-Kopfschmerzfragebogen, die einen Wechsel des Fragebogens unter Beibehaltung wichtiger Verlaufsparameter erlauben (Tab. 2). Auch eine Zusammenführung für wissenschaftliche Zwecke ist möglich.

**Tab. 2:** Vergleich DMKG-Kopfschmerzfragebogen und Deutscher Schmerzfragebogen (im Hinblick auf Erfassung von Kopfschmerzerkrankungen)

	DMKG-Kopfschmerzfragebogen	Deutscher Schmerzfragebogen
*(Kopf‑)Schmerzdaten*		
Schmerzstärke	Ja	Ja
Schmerzlokalisation	Zum Ankreuzen	Schmerzskizze
Kopfschmerztage/Monat	Ja	Nein
Schmerzmitteltage/Monat	Ja	Nein
Kopfschmerzcharakteristika	Ja	Teilweise^5^
Kopfschmerzkalender	Ja	Nur Tagesprotokoll
Bisherige kraniale Bildgebung	Ja	Nein
Auswirkungen im Alltag	Ja	Ja
Subjektive Schmerzursache	Nein	Ja
Verstärkende und lindernde Faktoren	Auslöser (im Kopfschmerzkalender)	Ja
*Behandlung*		
Aktuelle und frühere (Kopf‑)Schmerzmedikamente	Ja	Ja
Bisherige Schmerzbehandlungen: Verfahren und Operationen	Fokus auf nichtmed. Verfahren und Neurostimulation	Ja
Bisherige stationäre Schmerztherapien	Ja	Ja
Begleiterkrankungen + Begleitmedikation	Ja	Ja
Behandelnde Ärzt:innen mit Adressen	Nein	Ja
*Sonstiges*		
Demografische Daten	Ja	Ja
Sozialmedizinische Daten	Ja	Ausführlicher^1^
*Validierte Skalen*		
DASS	Ja	Ja
Gesundheitsbezogene Lebensqualität	Papier/PDF-Version: NRS^2^	VR-12
MFHW/FW‑7, SBL	Nein	Ja
MIDAS, aktueller Gesundheitszustand (NRS), PGIC, CHIQ	Ja^3,4^	Nein

^1^Zusätzlich: Frage nach offenen (versicherungs-) rechtlichen Fragen; Rentenantrag beabsichtigt/gestellt/abgelehnt/Widerspruch; detaillierte Erhebung der Krankenversicherung (Modul S und D)

^2^VR-12 nur in der geplanten digitalen Version, da händische Auswertung nicht möglich; in Papierversion Bewertung der gesundheitsbezogenen Lebensqualität durch Patient:in auf der NRS

^3^PGIC im Verlaufsbogen

^4^CHIQ im Zusatzmodul Clusterkopfschmerz

^5^Anhand Schmerzbeschreibungsliste (SBL, Korb 2006)

*MFHW* Marburger Fragebogen zum habituellen Wohlbefinden [16], *NRS* numerische Rating-Skala

## Einsatz des DMKG-Kopfschmerzfragebogens

Der DMKG-Kopfschmerzfragebogen dient als Grundlage für die Kopfschmerzanamnese. Bei der Erstvorstellung dient er auch der Erhebung eines Ausgangszustands für die spätere Verlaufsbeurteilung (mithilfe des Verlaufsbogens). Er stellt außerdem die Basis für eine interne und zentrenübergreifende Qualitätssicherung dar. Er ist kompatibel mit den Fragen bei Erstvorstellung und Wiedervorstellung im DMKG-Kopfschmerzregister. Ebenso wie das DMKG-Kopfschmerzregister erhebt der DMKG-Kopfschmerzfragebogen für die Anamnese wichtige demografische, sozialmedizinische und kopfschmerzbezogene Daten nach dem Prinzip der Datensparsamkeit.

Das Präsidium der DMKG hat am 27.09.2024 den DMKG-Kopfschmerzfragebogen für die Eingangserhebung und den DMKG-Verlaufsbogen für die Verlaufsbeurteilung von Kopfschmerzpatient:innen sowie das Clusterkopfschmerzzusatzmodul im Rahmen der Qualitätssicherungsvereinbarung Schmerztherapie (kbv.de/media/sp/Schmerztherapie.pdf) konsentiert. Der DMKG-Kopfschmerzfragebogen kann daher in diesem Zusammenhang zur Dokumentation genutzt werden.

## Nutzungsmöglichkeiten: Papierversion, ausfüllbares PDF und digitale Version

**Abb. 2:**
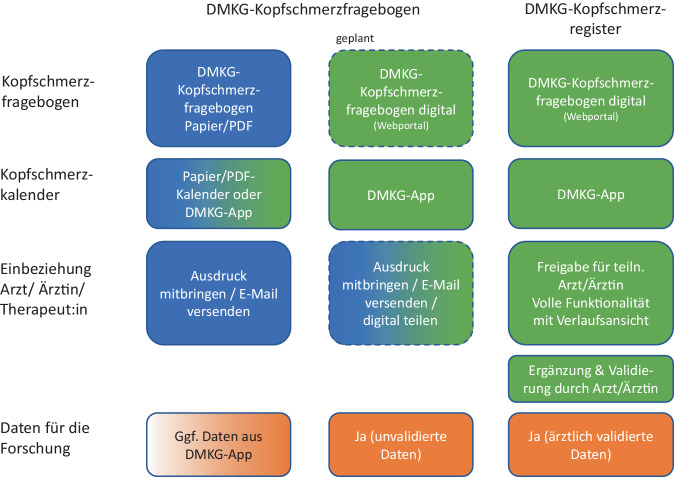
Illustration der Möglichkeiten der Nutzung des DMKG-Kopfschmerzfragebogens. *Blau* Papier/PDF-Version, *grün* digitale Version, *orange* Nutzung pseudonymisierter Daten für die Forschung. *Gestrichelter* Rand: in Entwicklung. (Für Details siehe Tab. 3)

Auch wenn eine digitale Erhebung Vorteile hat, ist diese nicht überall möglich oder gewünscht. Daher bietet die DMKG mehrere Möglichkeiten an, den DMKG-Kopfschmerzfragebogen zu nutzen und damit die Dokumentationsanforderungen der Qualitätsvereinbarung Schmerztherapie zu erfüllen (Abb. 2 und Tab. 3).

**Tab. 3:** Möglichkeiten der Anwendung des DMKG-Kopfschmerzfragebogens

	Versand an Patient:in	Versand von Patient:in an Arzt/Ärztin oder Therapeut:in	Vorteile	Kombination mit elektronischem Kopfschmerzkalender, z. B. DMKG-App
*Aktuell verfügbar*				
Papierversion	Download	Original mitbringen	Für jeden geeignet	Möglich. Report als PDF versenden oder ausgedruckt mitbringen
	Per E‑Mail	Scan per E‑Mail		
	Per Post	–		
Ausfüllbares PDF	Download	Per E‑Mail	Erhöhte Lesbarkeit, automatische Berechnung von Ergebnissen der Skalen, papiersparend	Möglich. Report als PDF versenden oder ausgedruckt mitbringen
	Per E‑Mail	Ausdruck mitbringen		
Kopfschmerzregister der DMKG	Patient:in erhält Web-Adresse^1^ und wird gebeten, sich zu registrieren und der Anleitung zu folgen	Freigabe für Arzt/Ärztin durch Patient:in, Ansicht im Arztportal	Automatische Validierung	Vorgesehen. Daten aus der DMKG-App werden automatisch in das Kopfschmerzregister übertragen
		PDF für Ablage herunterladbar	Komplette Eingaben	
			Umfangreichere Erhebung, z. B. Trennung in Akutmedikation und Prophylaxe, Wirksamkeit der Medikation, Nebenwirkungen	
			Automatische Einbindung des Kopfschmerzkalenders (DMKG-App)	
			Pseudonymisierte Daten für Kopfschmerzforschung (ärztlich validiert)	
			Übersichtliche Ansicht der Daten im Arztportal	
			Ergänzung und Validierung der Daten durch Arzt/Ärztin	
*In Planung*				
Kopfschmerzfragebogen digital	Patient:in erhält Web-Adresse und wird gebeten, sich zu registrieren und der Anleitung zu folgen. Patient:in erhält am Ende PDF-File mit Zusammenstellung der eingegebenen Daten	Per E‑Mail	Automatische Validierung	Empfohlen. Daten aus der DMKG-App werden automatisch an das PDF-File angehängt
		Ausdruck mitbringen	Komplette Eingaben	
		Digital teilen	Umfangreichere Erhebung, z. B. Trennung in Akutmedikation und Prophylaxe, Wirksamkeit der Medikation, Nebenwirkungen	
			Automatische Einbindung des Kopfschmerzkalenders (DMKG-App)	
			Pseudonymisierte Daten für Kopfschmerzforschung (nicht ärztlich validiert)	

^1^ www.kopfschmerzregister.de

### Papierversion/ausfüllbares PDF.

Die PDF-Version des DMKG-Kopfschmerzfragebogens ist für Fachkreise nach Registrierung frei verfügbar unter https://www.dmkg.de/dmkg-kopfschmerzfragebogen. Sie kann ausgedruckt und von Hand ausgefüllt werden. Alternativ kann sie als ausfüllbares PDF genutzt werden, und dem Arzt/der Ärztin ausgedruckt oder elektronisch zur Verfügung gestellt. Das ausfüllbare PDF verhindert (wo möglich) unplausible Eingaben und kann die Ergebnisse von DASS und MIDAS direkt berechnen und anzeigen. Dies kann sowohl mit der DMKG-App oder mit dem beigefügten PDF-Kopfschmerzkalender kombiniert werden. Im Verlauf ist die Übersetzung in weitere Sprachen geplant, wie dies schon beim DMKG-Kopfschmerzkalender erfolgreich umgesetzt wurde (mittlerweile in 12 Sprachen vorliegend, siehe www.dmkg.de).

### DMKG-Kopfschmerzfragebogen digital (in Planung).

Für die Zukunft geplant ist die Möglichkeit, dass Patient:innen den DMKG-Kopfschmerzfragebogen über eine Smartphone-App ausfüllen können und dort eine PDF-Version des ausgefüllten Bogens herunterladen. Diese kann dann ausgedruckt oder elektronisch an den Arzt/die Ärztin weitergegeben werden, ohne dass eine Teilnahme an der Vollversion des DMKG-Kopfschmerzregisters (siehe unten) notwendig ist. Die digitale Erhebung erlaubt eine detailliertere und (durch Verwendung von Drop-down-Menüs) validere Erfassung mancher Angaben, z. B. der Medikation. Skalen werden automatisch ausgewertet, inkomplette und unplausible Eingaben werden vermieden.

### DMKG-Kopfschmerzfragebogen digital innerhalb des DMKG-Kopfschmerzregisters.

Das Kopfschmerzregister der DMKG bietet zusammen mit der DMKG-App eine komplett digitale, seit 2020 etablierte Version des DMKG-Kopfschmerzfragebogens an. Das Kopfschmerzregister erfordert die aktive Mitarbeit der teilnehmenden Ärzt:innen, da Informationen ergänzt (z. B. die Kopfschmerzdiagnose) und zentrale Eingaben der Patient:innen validiert werden müssen. Die Teilnahme einer Praxis oder eines Zentrums am DMKG-Kopfschmerzregister erfordert eine DMKG-Mitgliedschaft und das individuelle DMKG-Kopfschmerzzertifikat. Weitere Informationen finden sich unter www.dmkg.de und www.kopfschmerzregister.de.

## Schlussfolgerung

Zusammenfassend ist der DMKG-Kopfschmerzfragebogen ein weiterer Baustein innerhalb des Angebots der DMKG zur Qualitätssicherung und Verbesserung der Kopfschmerzversorgung in Deutschland. Er ergänzt dabei die bestehenden Komponenten (AWMF-Leitlinien, DMKG-Kopfschmerzregister, DMKG-App, individuelles Kopfschmerzzertifikat der DMKG, Kopfschmerzzentrum-Zertifizierung der DMKG, umfangreiches Informationsmaterial für Ärzt:innen und Patient:innen zum Herunterladen oder kostenlosen Bestellen über die Awareness-Kampagne auf www.attacke-kopfschmerzen.de, Kopfschmerzkalender in vielen Sprachen auf www.dmkg.de).

## Fazit für die Praxis

- Der von Experten der DMKG entwickelte DMKG-Kopfschmerzfragebogen ist ein wichtiges Instrument zur Verbesserung und Qualitätssicherung der Kopfschmerzversorgung in Deutschland.
- Er soll von Patient:innen vorab ausgefüllt und in die Sprechstunde mitgebracht werden. Dort dient er als Basis für Anamnese und Verlaufsbeurteilung.
- Es gibt Versionen für Erst- und Wiedervorstellung sowie ein Clusterkopfschmerzmodul.
- Er kann als Papierversion oder als ausfüllbares PDF genutzt werden.
- Er kann mit dem beigefügten Papier/PDF-Kopfschmerzkalender oder mit der DMKG-App als elektronischem Kopfschmerzkalender kombiniert werden.
- Er steht für Fachkreise nach Registrierung kostenlos zum Download zur Verfügung.
- Eine digitale Version kann innerhalb des Projekts „DMKG-Kopfschmerzregister“ bereits genutzt werden, eine allgemein verfügbare digitale Version ist geplant.
